# Association Between Work‐Related Hyperthermia Emergency Department Visits and Ambient Heat in Five Southeastern States, 2010–2012—A Case‐Crossover Study

**DOI:** 10.1029/2019GH000241

**Published:** 2020-08-16

**Authors:** Jeffrey Shire, Ambarish Vaidyanathan, Michelle Lackovic, Terry Bunn

**Affiliations:** ^1^ National Institute for Occupational Safety and Health Centers for Disease Control and Prevention Cincinnati OH USA; ^2^ National Center for Environmental Health Centers for Disease Control and Prevention Atlanta GA USA; ^3^ Louisiana Public Health Institute New Orleans LA; ^4^ Kentucky Injury Prevention and Research Center, Department of Preventive Medicine and Environmental Health University of Kentucky College of Public Health Lexington KY USA

**Keywords:** heat stress disorders, ambient temperature, extreme heat, workers, case‐crossover, primary prevention

## Abstract

The objective of this study is to assess ambient temperatures' and extreme heat events' contribution to work‐related emergency department (ED) visits for hyperthermia in the southeastern United States to inform prevention. Through a collaborative network and established data framework, work‐related ED hyperthermia visits in five participating southeastern U.S. states were analyzed using a time stratified case‐crossover design. For exposure metrics, day‐ and location‐specific measures of ambient temperatures and county‐specific identification of extreme heat events were used. From 2010 to 2012, 5,017 work‐related hyperthermia ED visits were seen; 2,298 (~46%) of these visits occurred on days when the daily maximum heat index was at temperatures the Occupational Safety and Health Administration designates as having “lower” or “moderate” heat risk. A 14% increase in risk of ED visit was seen for a 1°F increase in average daily mean temperature, modeled as linear predictor across all temperatures. A 54% increase in risk was seen for work‐related hyperthermia ED visits during extreme heat events (two or more consecutive days of unusually high temperatures) when controlling for average daily mean temperature. Despite ambient heat being a well‐known risk to workers' health, this study's findings indicate ambient heat contributed to work‐related ED hyperthermia visits in these five states. Used alone, existing OSHA heat‐risk levels for ambient temperatures did not appear to successfully communicate workers' risk for hyperthermia in this study. Findings should inform future heat‐alert communications and policies, heat prevention efforts, and heat‐illness prevention research for workers in the southeastern United States.

## Key Points

- Workers being overexposed to heat has been shown to be a serious problem in the subtropical climate of the southeastern United States
- Almost half of the 5,017 work‐related emergency department hyperthermia visits in this study occurred on days with a heat index at “low” or “moderate” heat risk
- Appropriate heat‐alert thresholds and the timely and effective communication of heat risks to workers should be utilized to prevent workers from hyperthermia

## 1 Introduction

Hyperthermia is a known occupational condition that can lead to heat exhaustion, heat stroke (AFHSC, 2012; Baldwin & Hales, 2010, 2012; Brearley, 2016; Semenza et al., 1999; Stafoggia et al., 2008), and death (Arbury et al., 2014; Baldwin et al., 2014; Gubernot et al., 2015; Harduar Morano et al., 2016; Petitti et al., 2013).

Previously identified risk factors for work‐related hyperthermia include high temperature and humidity in the work setting, environmental (ambient) heat, physical exertion, heavy or protective clothing, exposure to direct sunlight, limited air movement, fluid loss, excessive perspiration, and a lack of heat acclimatization, work breaks, cooling areas, and adequate hydration (Jacklitsch et al., 2016; NIOSH, 1972, 1986a, 1986b). Workplace‐specific heat sources (e.g., furnaces, ovens, and structure/wildland fires) have also been identified as risk factors (Jacklitsch et al., 2016; NIOSH, 1972, 1986a).

Although a number of studies on the health effects of ambient heat on workers have been carried out in Australia (Brearley et al., 2013, 2015; Hanna et al., 2011; McInnes et al., 2018; Rameezdeen & Elmualim, 2017; Varghese, Barnett, et al., 2019; Varghese, Barnett, et al., 2019; Xiang et al., 2015), few studies have occurred in the United States. The need for heat morbidity studies has been particularly noted (Gubernot et al., 2015). Critical information is needed to successfully target prevention strategies for workers and track the effectiveness of existing or future prevention efforts. This need is especially great in the subtropical climate of the southeastern United States. During a 5‐year period (2007–2011), there were over 8,000 emergency department (ED) visits for work‐related hyperthermia in nine southeastern states (Harduar Morano et al., 2015). In a nationwide study of workers' mortality risk due to heat, five U.S. southeastern states (Arkansas, Florida, Mississippi, North Carolina, and South Carolina) were among the 10 U.S. states with the highest rates for occupational heat‐related deaths (Gubernot et al., 2015). A study of farmworkers conducted in Eastern North Carolina in 2013 found 72% of farmworkers experienced at least one heat‐related illness symptom during a work week (Kearney et al., 2016). Climate model simulations predict ambient heat and severe heat events will increase in southeastern United States in the coming decades (Carter et al., 2018).

To begin to characterize and better understand the specific risk factors for exposure of workers to heat in the southeastern United States, this study examines ED visits for work‐related hyperthermia in Florida, Georgia, Kentucky, Louisiana, and Tennessee between 2010 and 2012. These were the most recent years available at the study's onset. By linking these ED encounters to location‐ and day‐specific measures of ambient heat, we were able to evaluate the association between different temperature values and work‐related hyperthermia ED visits. These observations were also compared to current heat hazard guidelines as a frame of reference. This information can inform future prevention strategies and improve estimations of the burden of hyperthermia on the workforce in the southeastern United States.

## 2 Methods

### 2.1 Health Data and Case Definition

Based on a standard case definition of work‐related ED visits developed by the Southeastern Occupational Network (SouthON) (Harduar Morano et al., 2015), work‐related ED data from 2010 to 2012 were obtained from the health departments of the five southeastern U.S. states that were willing and able to participate in this study: Florida, Georgia, Kentucky, Louisiana, and Tennessee. The data were a census of ED visits from those states and were restricted to the months of May through September when temperatures are highest. To identify work‐related ED visits (i.e., workers), either (1) workers' compensation had to be the expected payer in the payment data field or (2) a work‐related external cause of injury code (Ecode) from ICD‐9‐CM (ICD‐9‐CM, Version 30, 2012) had to appear in any diagnosis or Ecode data field. These work‐related Ecodes were E000.0‐E000.1, E800‐E807 (fourth digit = 0), E830‐E838 (fourth digit = 2 or 6), E840‐E845 (fourth digit = 2 or 8), E846, and E849.1‐E849.3. From these work‐related ED visits, hyperthermia‐related cases were selected based on the presence of an ICD 9‐CM diagnosis or Ecode in any primary or secondary diagnosis data field: 992.0–992.9, E900.0, E900.1, or E900.9 (Table 1). Data were restricted to individuals aged 16 years and older to reflect the working‐age population. Hospital geographical location was coded to five‐digit Federal Information Processing Standards (FIPS) code of the county where the hospital was located. Collected demographic data on the ED encounters included age (years), sex, race (black, white, or other), and Hispanic ethnicity. Case‐address data were used to assign resident status (in‐state vs. out‐of‐state).

**Table 1:** Work‐Related Ecodes, Diagnosis Codes for Hyperthermia, and Ecodes for Hyperthermia

ICD‐9‐CM	Definition
E000.0	Civilian activity done for income or pay
E000.1	Military activity
E800‐E807	Railway accident among railway employee (fourth digit = 0)
E830‐E838	Water transport accident among crew, dockers, and stevedores (fourth digit = 2 or 6)
E840‐E845	Air and space transport accidents among crew and ground crew (fourth digit = 2 or 8)
E846	Accidents involving powered vehicles used within the buildings/premises of industrial or commercial establishment
992.0	Heat stroke and sunstroke
992.1	Heat syncope
992.2	Heat cramps
992.3	Heat exhaustion
992.4	Heat exhaustion due to salt depletion
992.5	Heat exhaustion, unspecified
992.6	Heat fatigue, transient
992.7	Heat edema
992.8	Other specified heat effects
992.9	Unspecified effects of heat and light
E900.0	Accident caused by excessive heat due to weather conditions
E900.1	Accident due to excessive heat of man‐made origin
E900.9	Accident due to excessive heat of unspecified origin

### 2.2 Ambient Heat Exposure Data

Hourly meteorological predictions from the North American Land Data Assimilation System Phase 2 (NLDAS) model (Mitchell et al., 2004), available at 0.125° grid resolution, were used to create measures of ambient heat exposures such as daily mean temperature and daily maximum heat index (HI). Hourly HI values were computed using hourly temperature and humidity information at the grid level, using a definition used by National Oceanic and Atmospheric Administration/National Weather Service (NWS, 2014). A multistage geo‐imputation approach (Fechter‐Leggett et al., 2016) was used to convert hourly grid‐level meteorological data to daily, county‐level estimates of mean temperature, and HI. Because the exact time of exposure within a given day was unknown for the ED encounters, we selected daily mean temperature (“Tmean”) to characterize daily synoptic heat exposure. Prior studies have noted the cumulative effects of heat exposure (Nag et al., 2013; Wichmann et al., 2013), so we created the primary exposure variable, “Average Tmean,” by averaging Tmean observed on the day of ED visit and three preceding days (aka a 3‐day “lag period” or “exposure offset”). In addition, extreme heat events (EHEs) pose a potential risk during summer months, and EHE definitions are available from literature. For this study an EHE was defined as a period of ambient heat exposure where daily, county‐level maximum HI values were greater than the 95th percentile county‐specific threshold for at least two consecutive days. Thus, each county had a single 95th percentile threshold value for maximum HI. This definition has been previously identified as one of the most appropriate definitions for EHEs in the southeastern United States (Vaidyanathan et al., 2016). Furthermore, county‐specific thresholds were estimated using summertime (May through September) HI data for a 30‐year period (1981–2010) to measure deviations and identify periods of extreme heat based on recent climate data. The EHE indicator (a binary indicator) was set to “1” if the day of ED visit and/or three preceding days were part of an EHE.

### 2.3 Statistical Analysis

Numerous recent studies have used the case‐crossover study design to examine the health effects associated with heat exposure (Liu et al., 2018; Lubczynska et al., 2015; McInnes et al., 2017, 2018; Sheffield et al., 2018; Sheng et al., 2018; Valent et al., 2016; Varghese, Barnett, et al., 2019; Varghese, Hansen, et al., 2019). This case‐crossover study design uses only “cases” for the health outcome matched with “exposures” for the case day and control day(s). For this study, a time‐stratified case‐crossover study design (Janes et al., 2005) was used to model the relationship between heat exposure variables and encounter‐level work‐related hyperthermia ED visits. In the time‐stratified case‐crossover design, each exposure value for an index date (i.e., ED visit date) is compared to control days that are matched to the index date based on the same day of the week within a time period. Specifically, each month was a priori divided into two periods, (i) Days 1–15 and (ii) Days 16 to end of month, and control day(s) were selected on the same day of the week as the case within the same half of the month (Saha et al., 2015). This method has been shown to accurately control for time‐invariant confounding variables documented for each case (e.g., worker's age, gender, and occupation) and reduce bias from seasonal time trends in the exposure and time‐varying factors (e.g., day of the week) (Janes et al., 2005; Lumley & Levy, 2000). We implemented this study design using conditional logistic regressions to calculate mean odds ratios (ORs) with 95% confidence intervals (CI) to evaluate associations between measures of ambient heat exposure and work‐related hyperthermia visits. We calculated ORs for the average daily mean temperatures as a predictor for work‐related hyperthermia ED visits by month and also assessed the ORs stratified by lag days out to 6 days prior to the day of the ED visit. Both the Average Tmean and the EHE indicator were included as independent variables to calculate the ORs in stratified analyses of the individual covariates (e.g., age and sex). We also calculated ORs for the Average Tmean and the EHE indicator by state. All primary statistical analyses were performed with SAS v9.4 (SAS Institute Inc., 2019).

## 3 Results

During the 3‐year study period, there were 5,017 work‐related hyperthermia ED visits for the five southeastern states. Of the total visits, 90% of the work‐related hyperthermia emergency visits were in‐state residents, and 87% were males (Table 2). The most frequently treated age groups treated in the ED for hyperthermia were younger workers: 20–24 years (13%), 25–29 years (14%), and 30–34 years (14%). Sixty‐nine percent of the ED visits for work‐related hyperthermia were for the non‐Hispanic ethnicity group; only 6% were Hispanic workers—although the ethnicity was unavailable in 25% of the ED visits for hyperthermia. Most of the hyperthermia ED visits were coded as white workers (72%); 22% were black.

**Table 2:** Demographic Characteristics of Work‐Related Hyperthermia Emergency Department Visits in Five Southeastern States, 2010–2012

Characteristic	Frequency	Percentage
State Resident Status		
Non‐state resident	487	10
Unknown	5	<1
State resident	4,525	90
Sex		
Female	641	13
Male	4,376	87
Age		
16–19	198	4
20–24	672	13
25–29	715	14
30–34	692	14
35–39	610	12
40–44	608	12
45–49	570	11
50–54	458	9
55–59	279	6
60–64	142	3
65+	73	1
Ethnicity		
Hispanic	295	6
Non‐Hispanic	3,471	69
Unavailable	1,251	25
Race		
Black	1,081	22
White	3,574	71
Other	282	5
Unavailable	80	2
Total	5,017	100

**Figure 1:**
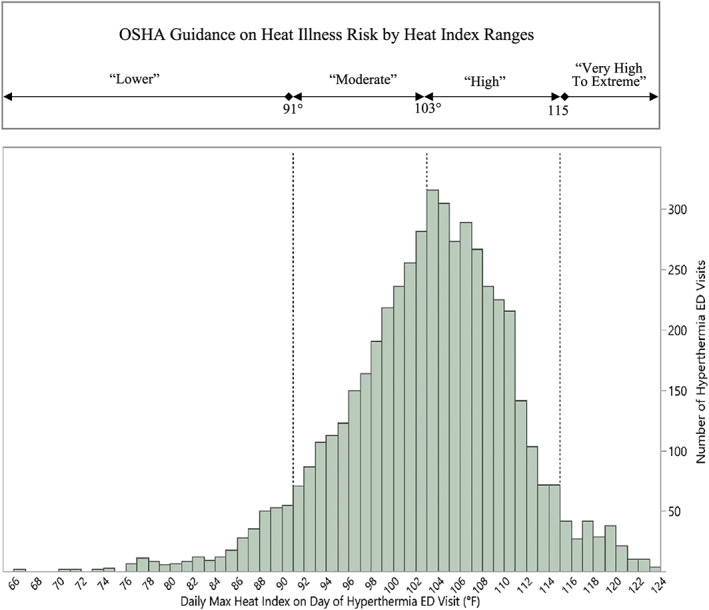
Comparison of OSHA heat risk levels and heat index of emergency department visits. (a) OSHA heat risk levels by temperature range. (b) Work‐related hyperthermia emergency department visit frequencies by daily maximum heat Index, five southeastern U.S. states, 2010–2012 (*N* = 5,017).

Forty‐six percent of the hyperthermia ED visits occurred on days when the daily maximum HI was less than 103°F—which includes the temperatures the Occupational Safety and Health Administration (OSHA) designates as having “lower” (HI < 91°F) or “moderate” heat risk (91°F <HI <103°F) (OSHA, 2019; Figure 1). The number of hyperthermia ED visits peaked when the daily HI value was 103°F. The median value for daily HI temperatures was 104°F for all work‐related hyperthermia ED visits in this study.

The unstratified logistic regression modeling results showed an approximately 14% increase in the risk of a work‐related hyperthermia ED visit for a 1°F increase in average daily mean temperature (Average Tmean, i.e., the combined average temperature for the day of ED visit and the three preceding days) when controlling for days within an EHE (Table 3). Average Tmean was also shown to be a significant predictor of work‐related hyperthermia ED visits when stratified for age, sex, ethnicity, race, and residency status.

**Table 3:** Odds Ratios of Average Daily Mean and Extreme Heat Indicator as Predictors for Work‐Related Hyperthermia Emergency Department Visits, Stratified by Demographics

Stratification level	Covariate category	Model predictors^a^			
		Average daily mean temperature (°F)		EHE indicator	
		Odds ratio (95% CI)	*p* value	Odds ratio (95% CI)	*p* value
No strata	All	1.136 (1.115, 1.158)	<0.0001	1.536 (1.384, 1.705)	<0.0001
Age	(16–34)	1.138 (1.108, 1.169)	<0.0001	1.631 (1.396, 1.905)	<0.0001
	(≥35)	1.135 (1.106, 1.165)	<0.0001	1.464 (1.272, 1.686)	<0.0001
Gender	Female	1.113 (1.061, 1.166)	<0.0001	1.981 (1.468, 2.673)	<0.0001
	Male	1.140 (1.117, 1.164)	<0.0001	1.481 (1.324, 1.655)	<0.0001
Ethnicity	Hispanic	1.136 (1.030, 1.252)	0.0109	1.152 (0.755, 1.757)	0.5127
	Non‐Hispanic	1.141 (1.117, 1.166)	<0.0001	1.494 (1.322, 1.688)	<0.0001
Race	Non‐White	1.154 (1.112, 1.197)	<0.0001	1.495 (1.228, 1.821)	0.0001
	White	1.130 (1.106, 1.155)	<0.0001	1.551 (1.372, 1.755)	<0.0001
Residency	Nonresident	1.143 (1.076, 1.213)	<0.0001	1.864 (1.333, 2.607)	0.0003
	Resident	1.136 (1.113, 1.158)	<0.0001	1.504 (1.348, 1.679)	<0.0001

*Note*. Average daily mean temperature (°F) was modeled as a 1° change. The Average Tmean variable is calculated as an average of temperatures observed on the day of ED visit and three preceding days for the county where the ED visit occurred. EHE indicator is a binary indicator and is set to “1” if the day of the ED visit or three preceding days were part of an extreme heat event.

^a^ All estimates of odds ratios include both “Average Tmean” and “EHE Indicator” as independent variables in the logistic regression models.

**Figure 2:**
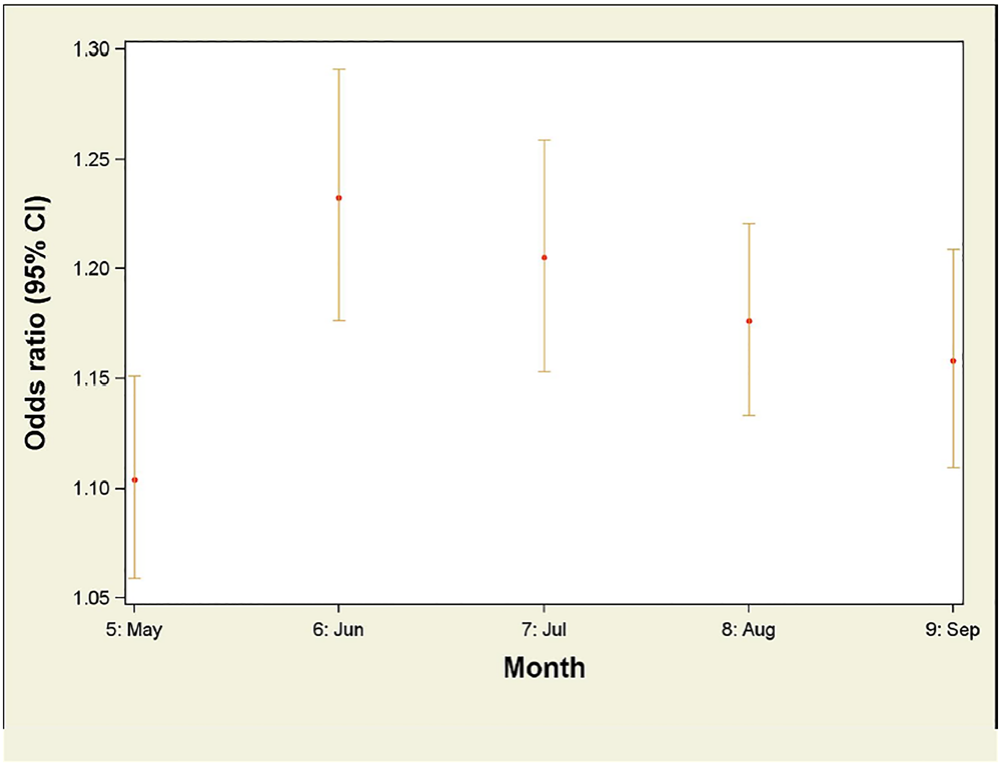
Odds ratios for average daily mean temperatures as a predictor for work‐related hyperthermia emergency department visits, stratified by month.

**Figure 3:**
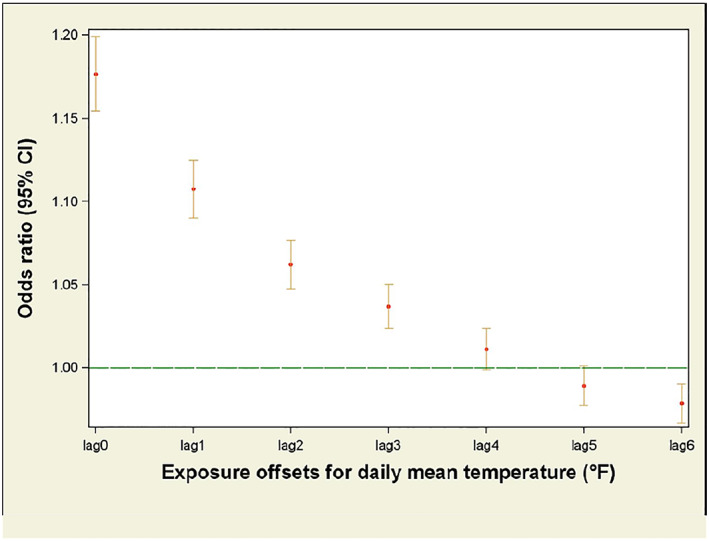
Odds ratios of daily mean temperatures as a predictor for work‐related hyperthermia emergency department visits, stratified by lag days. A lag period accounts for delayed or extended health effects associated with an exposure. Odds ratios for lag days 1–3 were elevated (i.e., >1); lag days 4–6 were not. Lag day 0 is the day of visit.

For associations between Average Tmean and work‐related hyperthermia ED visits when stratified by month, the mean work‐related hyperthermia risk increased from May to June (Figure 2). ORs associated with temperature were highest on the day of the ED encounter; however, ORs remained statistically significant for a lag period of up to 3 days (Figure 3).

An approximately 54% increase in risk was seen for work‐related hyperthermia ED visits during an EHE when controlling for Average Tmean. EHE events were also significantly associated with work‐related hyperthermia ED visits when stratified by age, sex, race, and residency and controlling for Average Tmean. However, days within an EHE were not associated with ED visits for Hispanic workers when controlling for Average Tmean; EHE events were associated with ED visits for work‐related hyperthermia for non‐Hispanic workers. State‐specific ORs for Average Tmean were similar (Table 4). The ORs for EHEs by state were significantly associated with work‐related hyperthermia ED visits for four states but not for Florida.

**Table 4:** Odds Ratios of Average Daily Mean and Extreme Heat Indicator as Predictors for Work‐Related Hyperthermia Emergency Department Visits, by State

No strata				
All 5 states	1.136 (1.115, 1.158)	<0.0001	1.536 (1.384, 1.705)	<0.0001
Florida	1.143 (1.087, 1.201)	<0.0001	1.135 (0.920, 1.401)	0.2363
Georgia	1.098 (1.047, 1.152)	<0.0001	1.723 (1.372, 2.163)	<0.0001
Kentucky	1.160 (1.114, 1.209)	<0.0001	2.164 (1.567, 2.988)	<0.0001
Louisiana	1.117 (1.071, 1.165)	<0.0001	1.847 (1.449, 2.353)	<0.0001
Tennessee	1.143 (1.103, 1.184)	<0.0001	1.453 (1.172, 1.802)	<0.0001

Average daily mean temperature (°F) was modeled as a 1‐degree change. The Average Tmean variable is calculated as an average of temperatures observed on the day of ED visit and three preceding days for the county where the ED visit occurred. EHE indicator is a binary indicator and is set to “1” if the day of the ED visit or three preceding days were part of an extreme heat event. All estimates of odds ratios include both “Average Tmean” and “EHE Indicator” as independent variables in the logistic regression models.

## 4 Discussion

This study's findings indicate ambient heat contributed to work‐related ED hyperthermia visits in the five southeastern states for the period of 2010 to 2012. A 1°F increase in average daily mean temperature was associated with a 14% increase in risk of a work‐related hyperthermia ED visit. Additionally, there was a 54% increase in risk for a work‐related hyperthermia ED visit during an EHE. A large percentage of work‐related hyperthermia ED visits seen in this study occurred on days when the HI was at temperatures OSHA designates as having a “lower” or “moderate” risk.

Heat‐related illness in the workplace is a well‐recognized problem, and appropriate prevention measures have been well documented (California, 2015; Jacklitsch et al., 2016; NIOSH, 1972, 1986a; Washington, 2008). These measures primarily focus on recognizing risks, minimizing fluid and electrolyte depletion, ensuring appropriate intake of fluids, and monitoring level of work activity that can be performed safely in work environments with elevated temperatures. Despite established prevention measures, our study shows that workers continued to be overexposed to heat. Our paper is the only analysis we are aware of that assesses work‐related hyperthermia cases treated in EDs against day‐ and location‐specific measures of ambient temperatures and EHEs in the United States. The value of using spatially and temporally specific data was demonstrated in our finding of a significant OR for a 3‐day lag time.

The results of our study are similar to what have been found in other studies: Studies of workers' compensation claims for heat‐illness in Australia found a 12.7% increase in claims per 1°F increase in daily mean temperature (Varghese, Barnett, et al., 2019; Xiang et al., 2015). While some of our findings reinforced what has been observed in previous research, an important and unique finding from our analysis was the statistically significant ORs observed for a lag period of up to 3 days. These results indicate that work‐related hyperthermia ED visits were associated with ambient heat from up to 3 days prior to the ED visit and suggest that a recovery period may have been critical during periods of extreme heat stress. Such recovery periods may be important, especially in occupations employing workers whose access to air conditioning may be limited (Kearney et al., 2016).

In this study we observed a decline in the number of work‐related hyperthermia ED visits as temperatures rose above a maximum daily HI of 103°F (Figure 1). One possible explanation for this is employers and workers may have recognized an obvious heat risk at these temperatures and higher and therefor implemented heat‐safety practices and policies.

Although EHEs were a significant predictor for race, sex, and age, Hispanic ethnicity was not associated with EHEs within this study. This finding has several important caveats. Only 93 hyperthermia ED visits appeared for Hispanics during an EHE within this study, and the CI for the estimated OR was relatively large. Additionally, only 6% of the ED visits for hyperthermia in this study were Hispanic workers while an estimated 12% of all workers (in full‐time equivalents) were Hispanic in these five states in 2011(BLS, 2011; NIOSH, 2019). Previous studies have shown that access to healthcare can be a barrier for some Hispanic working populations (Frank et al., 2013). However, our study is made up of only those individuals who obtained healthcare at an ED and did not include those who either did not seek treatment or sought treatment at a non‐ED facility. This means that this study is only able to assess the relationship between heat exposure and ED visit for those Hispanic workers who sought treatment at an ED. We had no additional information to determine whether that relationship was biased or was unbiased. Ideally, these findings will be addressed in future studies that will include more hyperthermia ED visits for Hispanics during EHEs and include cases from other treatment centers such as urgent care centers, migrant health clinics, and community health clinics.

The apparent changes in heat risk observed across months (Figure 2) could reflect underlying awareness or prevention processes occurring. Employers' and workers' awareness of the heat‐illness risk of ambient temperatures could have increased as temperatures rose during the early part of summers then leading to utilizing heat‐illness prevention measures. Workers may have become better acclimated to heat over the course of the summers, and/or there could have been a kind of survivor effect in which workers who were more prone to heat illness changed jobs or work tasks having less exposure to heat. Perhaps, there was a nonlinearity in the risk of hyperthermia ED visits across the full spectrum of ambient temperatures.

This analysis was only possible through a unique network of state public health agencies and Centers for Disease Control and Prevention (CDC) researchers that facilitated access to multistate, multiyear data and leveraged advanced data analytic approaches. This collaborative network and established data framework have the potential for further advancing the use of local data to better assess and inform worker health issues. Importantly, this research was coordinated through a regional occupational health network of southeastern states (Southeastern Occupational Network [SouthON]), and researchers at CDC's National Institute for Occupational Safety and Health (NIOSH) and the National Center for Environmental Health. This network integrates occupational safety and health capacity, practice, and research at state and regional levels by fostering collaborations among state health department staff and external partners such as CDC, OSHA, and universities.

Our study combined two unique data sets: ED visits and weather data. The ED data included over 5,000 unique records of work‐related ED encounters from five states for a 3‐year period. It is important to note that locational information at a geographic resolution below state level is often excluded from large, publicly available data sets. For example, the National Center for Health Statistics National Inpatient Hospitalization and National Emergency Department data sets are limited to analysis at the regional or national level, which limits evaluating intrastate and interstate weather variations within regions for targeting outreach and prevention activities, including weather alerts. The daily, county‐level weather data used for this analysis provide a firm basis to account for spatiotemporal variation across counties and days for heat exposure. The ED visit data were only available to us at the county level, but to attempt to account for potential within county variations of temperatures, we used a population‐weighted average for a given day for all grid cells within each county. Further, the thresholds used to define EHEs were location specific and were derived using a long‐term time series of daily maximum HI values. Hence, increases in hyperthermia risks during EHEs can be interpreted from a climatological perspective, which is an important consideration to devise adaptation strategies for mitigating work‐related heat exposures under various climate change scenarios.

In addition, using fine‐scale data to address workers' health issues can help create various heat intervention strategies that are attuned to locally relevant health risks and help inform policies and applications necessary to mitigate negative health outcomes for workers. For instance, OSHA and CDC have partnered to offer the “OSHA‐NIOSH Heat Safety Tool” mobile app for iOS and Android devices (NIOSH, 2017). This mobile app determines HI values—a measure for how hot it feels—based on the current location‐specific temperature and humidity data from the U.S. National Weather Service. In the future, such collaborations can provide a platform to evaluate the alignment between heat alert thresholds used by the National Weather Service and OSHA and the observed health burden within various alert ranges.

### 4.1 Limitations

There are at least six limitations to this study. First, we used medical billing data that were not originally collected for epidemiological analyses. Second, our case definition of work‐related ED encounters for hyperthermia could be subject to systematic bias(es) in identifying work relatedness and/or “hyperthermia.” Payment source is commonly used as a proxy to identify work‐related medical encounters, but that approach undercounts workers because workers' compensation is underutilized and has been shown to miss up to 47% of work‐related injuries (Groenewold & Baron, 2013). We used Ecodes to help address this known issue. Third, exposure misclassification may have occurred as a result of using the location of the ED facility to link to the appropriate location information on ambient temperatures. Although ambient temperatures tend to be highly autocorrelated spatially, there exists the possibility that the measure of “exposure” data used in our analysis could differ from those temperatures, both ambient and indoors, experienced by the workers. Fourth, we had no information on workers' occupations, work tasks, acclimatization to heat, or new‐worker status—all of which are important risk factors for heat stress. Fifth, although the methods used in this case‐crossover analysis have been widely used and accepted within the field of epidemiology, there remains the possibility that some bias for the control days relative to the case days could be present in these data. Finally, because the case‐crossover design uses only cases, valid inferences can be made about the relationship between the cases and the exposures—not about the prevalence of the cases or direct comparisons between demographics of cases, and so forth. Because of this, we have not attempted to interpret any variations observed in the prevalence of cases across the various demographics. This is also why no demographics were included as predictors in any of the conditional logistic regression models because case demographics remain constant for each case event. Instead, we used stratified analyses to examine the ORs for ambient temperatures and EHEs for different demographic strata.

## Conflict of Interest

The authors declare no conflict of interest relevant to this study.

## Supporting information

Supporting Information S1

Table S1

## Data Availability

The North American Land Data Assimilation System Phase 2 (NLDAS) model data can be obtained via NASA's Goddard Earth Sciences Data and Information Services Center (https://disc.gsfc.nasa.gov/). The emergency department data used in this study were obtained via state health departments' data use agreements with the data steward for each state's emergency medical records and are not accessible to the public or research community. The Health Insurance Portability and Accountability Act precludes distribution of the health outcome data used in this analysis.
