# Supplementary material for: Association Between Work‐Related Hyperthermia Emergency Department Visits and Ambient Heat in Five Southeastern States, 2010–2012—A Case‐Crossover Study
Source: Geohealth. 2020 Aug 16;4(8):e2019GH000241. doi: 10.1029/2019GH000241 (PMC7429406; doi:10.1029/2019GH000241)
Supplement: Supplementary file 1 — Supporting Information S1 [file GH2-4-e2019GH000241-s001.docx]

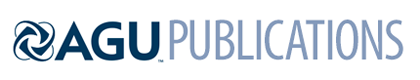


*GeoHealth*

Supporting Information for

**Association between Work-Related Hyperthermia Emergency Department Visits and Ambient Heat in Five Southeastern States, 2010-2012 —a Case-Crossover Study**

**Jeffrey Shire^1^†, Ambarish Vaidyanathan^2^†, Michelle Lackovic^3^, Terry Bunn^4^**

^1^National Institute for Occupational Safety and Health, Centers for Disease Control and Prevention, Cincinnati, OH,

^2^National Center for Environmental Health, Centers for Disease Control and Prevention, Atlanta, GA,

^3^Louisiana Public Health Institute, New Orleans, LA,

^4^Kentucky Injury Prevention and Research Center, Department of Preventive Medicine and Environmental Health, University of Kentucky College of Public Health, Lexington, KY

**Contents of this file**

Figure S1: Map of the 95th Percentile County-specific Thresholds for Daily Maximum Heat Index During Summertime (May through September) for a 30-year period (1981 – 2010) for Participating Southeastern Occupational Network States.

Figure S2: Results of Sensitivity Analysis, Odds Ratios for Work-related ED Visits for Hyperthermia to Type by Daily Heat Metrics

Tables S1: Cross-tabulation of Work-related ED Cases for Hyperthermia, State by Month

**Additional Supporting Information** (uploaded separately)

Caption for Table S2: 95th Percentile Thresholds for Daily Maximum Heat Index During Summertime (May through September) for a 30-year period (1981 – 2010) for Participating SouthON Counties—listed by five-digit Federal Information Processing Standard (FIPS5) codes.

**Introduction**

What follows are supplemental figures and tables that, though not central to this manuscript, may be of interest to others.


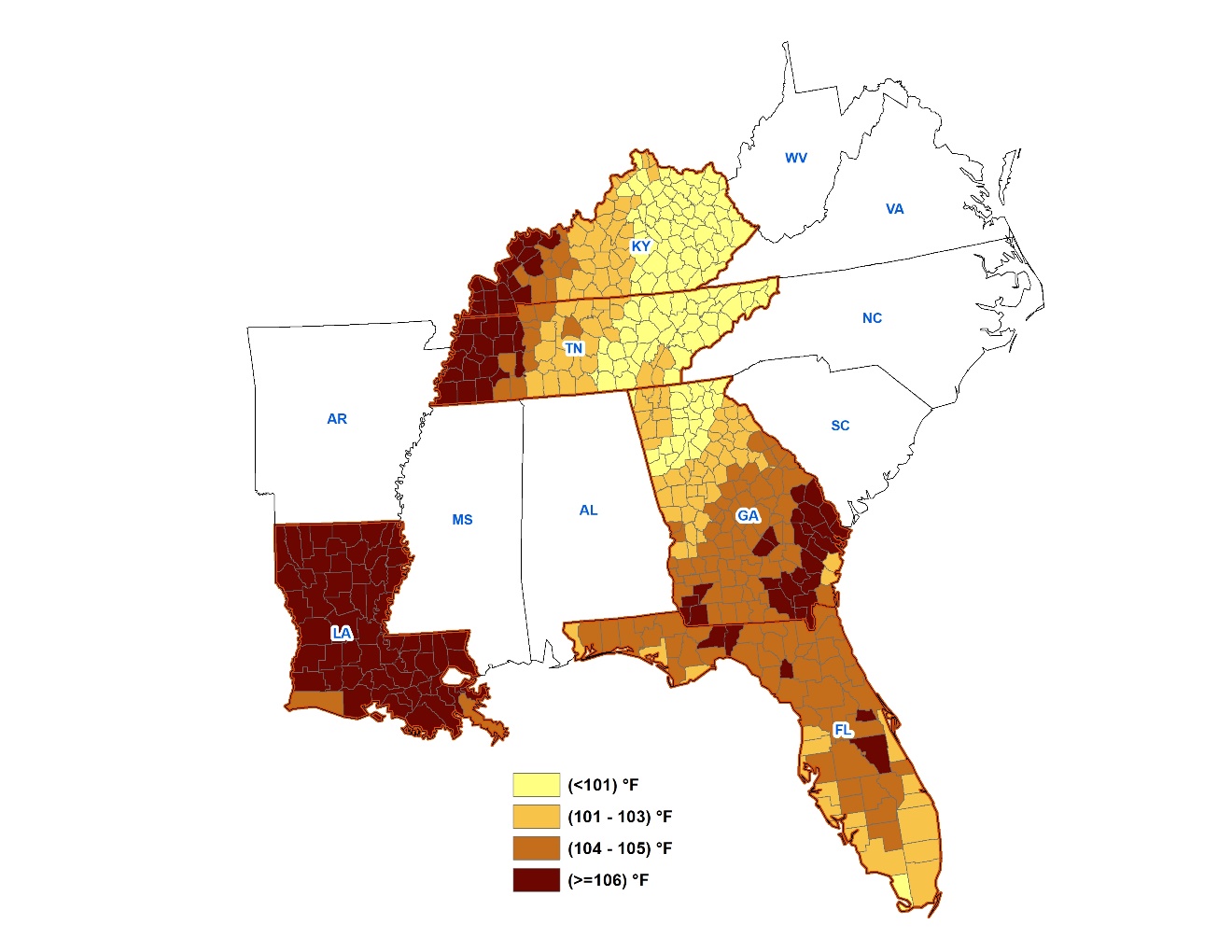


**Figure S1**: Map of the 95^th^ Percentile County-specific Thresholds for Daily Maximum Heat Index During Summertime (May through September) for a 30-year period (1981 – 2010) for Participating Southeastern Occupational Network States.

**Figure S2**: Results of Sensitivity Analysis, Odds Ratios for Work-related ED Visits for Hyperthermia to Type by Daily Heat Metrics

|  | | |  |  |  |  |  |  |  |  |
| --- | --- | --- | --- | --- | --- | --- | --- | --- | --- | --- |
|  |  |  |  |  |  |  |  |  |  |  |
| **Work-related ED Cases for Hyperthermia, State by Month** | | | | | | | | | | |
| **State** | | | | | **Month** | | | | | |
| Frequency Row Percentage Column Percentage | | | | | **May** | **June** | **July** | **August** | **September** | **Total** |
| **FL** | | | | | 157 11.57 43.37 | 306 22.55 23.98 | 376 27.71 23.28 | 397 29.26 27.76 | 121 8.92 36.23 | 1357 |
| **GA** | | | | | 51 6.26 14.09 | 254 31.17 19.91 | 280 34.36 17.34 | 190 23.31 13.29 | 40 4.91 11.98 | 815 |
| **KY** | | | | | 36 5.86 9.94 | 121 19.71 9.48 | 275 44.79 17.03 | 150 24.43 10.49 | 32 5.21 9.58 | 614 |
| **LA** | | | | | 71 6.21 19.61 | 308 26.92 24.14 | 286 25.00 17.71 | 380 33.22 26.57 | 99 8.65 29.64 | 1144 |
| **TN** | | | | | 47 4.32 12.98 | 287 26.40 22.49 | 398 36.61 24.64 | 313 28.79 21.89 | 42 3.86 12.57 | 1087 |
| **Total** | | | | | 362 | 1276 | 1615 | 1430 | 334 | 5017 |

**Table S1**: Cross-tabulation of Work-related ED Cases for Hyperthermia, State by Month. Resulting cells show the count (frequency), row-percentage, and column-percentage for that cell.
